# Bioavailable Phenolic Compounds from Olive Pomace Present Anti-Neuroinflammatory Potential on Microglia Cells

**DOI:** 10.3390/foods12224048

**Published:** 2023-11-07

**Authors:** Luana Schmidt, Bruna Krieger Vargas, Camila Sant’Anna Monteiro, Lauren Pappis, Renius de Oliveira Mello, Alencar Kolinski Machado, Tatiana Emanuelli, Marco Antônio Zachia Ayub, José Cláudio Fonseca Moreira, Paula Rossini Augusti

**Affiliations:** 1 Institute of Basic Health Sciences, Postgraduate Program in Biological Sciences: Biochemistry, Federal University of Rio Grande do Sul (UFRGS), Ramiro Barcelos Street, 2600-Annex, Porto Alegre CEP 90035-003, RS, Brazil; luana.schmidt020@gmail.com (L.S.); 00006866@ufrgs.br (J.C.F.M.); 2 Institute of Food Science and Technology, Federal University of Rio Grande do Sul (UFRGS), Av. Bento Gonçalves, 9500, Campus do Vale, Porto Alegre CEP 91501-970, RS, Brazilmazayub@ufrrgs.br (M.A.Z.A.); 3 Department of Food Technology and Science, Center of Rural Sciences, Federal University of Santa Maria, Camobi, Santa Maria CEP 97105-900, RS, Brazil; 4 Graduate Program in Nanoscience, Franciscan University, Santa Maria CEP 97105-900, RS, Brazil; 5 Laboratory of Cell Culture and Genetics, Franciscan University, Santa Maria CEP 97105-900, RS, Brazil

**Keywords:** neuroinflammation, oxygen reactive species, nitric oxide, gastrointestinal digestion, BV-2 cells

## Abstract

The neuroinflammatory process is considered one of the main characteristics of central nervous system diseases, where a pro-inflammatory response results in oxidative stress through the generation of reactive oxygen and nitrogen species (ROS and RNS). Olive (*Olea europaea* L.) pomace is a by-product of olive oil production that is rich in phenolic compounds (PCs), known for their antioxidant and anti-inflammatory properties. This work looked at the antioxidant and anti-neuroinflammatory effects of the bioavailable PC from olive pomace in cell-free models and microglia cells. The bioavailable PC of olive pomace was obtained through the process of in vitro gastrointestinal digestion of fractionated olive pomace (OPF, particles size < 2 mm) and micronized olive pomace (OPM, particles size < 20 µm). The profile of the PC that is present in the bioavailable fraction as well as its in vitro antioxidant capacity were determined. The anti-neuroinflammatory capacity of the bioavailable PC from olive pomace (0.03–3 mg L^−1^) was evaluated in BV-2 cells activated by lipopolysaccharide (LPS) for 24 h. The total bioavailable PC concentration and antioxidant activity against peroxyl radical were higher in the OPM than those observed in the OPF sample. The activation of BV-2 cells by LPS resulted in increased levels of ROS and nitric oxide (NO). The bioavailable PCs from both OPF and OPM, at their lowest concentrations, were able to reduce the ROS generation in activated BV-2 cells. In contrast, the highest PC concentration of OPF and OPM was able to reduce the NO levels in activated microglial cells. Our results demonstrate that bioavailable PCs from olive pomace can act as anti-neuroinflammatory agents in vitro, independent of particle size. Moreover, studies approaching ways to increase the bioavailability of PCs from olive pomace, as well as any possible toxic effects, are needed before a final statement on its nutritional use is made.

## 1. Introduction

Neuronal diseases associated with neurodegeneration and neuroinflammation are reported as a public health problem worldwide [1]. Alzheimer’s disease is the most common neurodegenerative disease and accounts for 60 to 70% of all dementia cases, followed by Parkinson’s disease [2,3]. It is estimated that approximately 50 million individuals worldwide are afflicted by dementia, a figure that is projected to rise to 130 million by 2050 [3].

The neuroinflammation process is considered one of the main mechanisms of the central nervous system (CNS) for dealing with pathogenic agents or situations of impairment in cellular physiology, and it is associated with the activation of microglia cells, which are considered the immune cells of the CNS [4,5]. The activation of microglial cells initiates an immune response to injuries or diseases in the central nervous system, characterized by an increased production of pro-inflammatory cytokines [1,5].

The production of pro-inflammatory cytokines and chemokines induced by activated microglia cells results in an increase in oxidative stress through the generation of reactive oxygen and nitrogen species (ROS and RNS) [6,7]. Oxidative stress has been associated with several diseases, including neuropsychiatric and neurodegenerative conditions, most of which present chronic immune activation [7,8]. In fact, the inflammatory inducers phytohemagglutinin (PHA) and lipopolysaccharide (LPS) are capable of inducing ROS and RNS generation along with cell inflammation in murine macrophages and microglia cells, respectively [1,6].

In this context, it is important to seek therapeutic strategies through the anti-neuroinflammatory capacity that can modify the polarization of activated microglia cells, in which the pro-inflammatory pathway is their main immune response [1]. Thus, natural compounds capable of modulating the immune response of activated microglia cells and/or reducing oxidative stress seem to be a promising alternative [1].

PCs are widely produced by plants, such as berries, nuts, and olives, and are known to present antioxidant and anti-inflammatory activities, especially in models associated with the CNS [2,5]. Besides regular sources, PCs can be obtained from waste and by-products from the food industry, which has been a sustainable and great alternative for obtaining these compounds [6].

Olive (*Olea europaea* L.) pomace is a by-product of olive oil production, consisting of pulp, peel, and olive pits and representing about 80% (*v*/*v*) of the total amount of processed olives. Olive pomace has a considerable amount of PCs (0.4 to 2.4%) [9]. Due to their hydrophilic characteristics, 98% of the PCs present in the olive migrates to the pomace fraction during olive oil extraction [10]. Additionally, PCs from olive fruits, such as hydroxytyrosol and oleuropein, present antioxidant and anti-inflammatory effects in microglia cells activated by lipopolysaccharide (LPS), a known model for inflammation [11,12,13].

There is an abundance of PCs in the olive pomace, which makes it extremely promising for the food industry, and processes that increase the bioavailability of these compounds are extremely important [14]. Particle size reduction processes, such as micronization, are capable of increasing the intestinal bioavailability of olive pomace PCs after in vitro digestion [9]. However, throughout the digestive process, structural changes occur in PCs due to the action of enzymes and intestinal microbiota [15]. The process of digestion of olive pomace contributes to the formation of PCs derived from oleuropein, tyrosol, and hydroxytyrosol, which have a greater bioavailability [9]. Depending on the dose, hydroxytyrosol and tyrosol have the highest intestinal absorption rates (40–60%). Secoiridoid compounds are stable in the mouth, but significant losses occur in the gastric, duodenal, and intestinal regions due to the degradation of oleuropein and the formation of tyrosol and hydroxytyrosol [9,14].

Therefore, olive pomace PCs are promising compounds for the food industry and have different biological properties, such as antioxidant and anti-neuroinflammatory potential [14]. Thus, it is important to evaluate the beneficial potential of the bioavailable fraction of PC in foods before digestion processes. Based on these considerations, the aims of this work were to evaluate the antioxidant activity in cell-free models and the anti-neuroinflammatory capacity of bioavailable PCs from olive pomace on activated microglia cells.

## 2. Material and Methods

### 2.1. Olive Pomace Samples

Samples of olive pomace, cv. ‘Arbequina’, were obtained from an olive oil extractor company located in the city of Formigueiro, RS, Brazil (29°59′01″ S; 53°21′50″ W) and were processed according to the methodology described by [16]. The OP without physical modification was freeze-dried in a freeze dryer (LS 3000, Terroni Equipments Cientifiques, São Paulo, Brazil) and crushed in a knife mill (MA 630, Marconi^®^, São Paulo, Brazil). The raw OP was subjected to granulometry fractionation with a 2 mm sieve and centrifuged (1774× *g* for 10 min). The sediment was collected, lyophilized in a lyophilizer (LS 3000, Terroni Equipments Scientific, São Paulo, Brazil), crushed in a food mill knife (MA 630, Marconi^®^, SP, Brazil), and degreased with n-hexane. The fraction obtained was called OPF. Subsequently, the OPF was micronized in a planetary ball mill (PM 100, Retsch Co., Haan, Germany), using a 250 mL container with six stainless steel spheres (30 mm in diameter each). The grinding time was optimized for 15 g of sample ground at 300 rpm min^−1^ for 5 h, with a 2 min pause every 10 min of grinding. After micronization, the sample was named OPM. In this way, the raw OP sample resulted in two samples with different particle sizes: (a) OPF—Olive pomace obtained via moist fractionation in a strainer (particles < 2 mm) and lyophilized, milled in a common knife mill, and degreased; and (b) OPM—Olive pomace obtained as described above and micronized into particles < 20 µm.

### 2.2. Gastrointestinal Digestion of Olive Pomace to Obtain Bioavailable PCs

**Figure 1:**
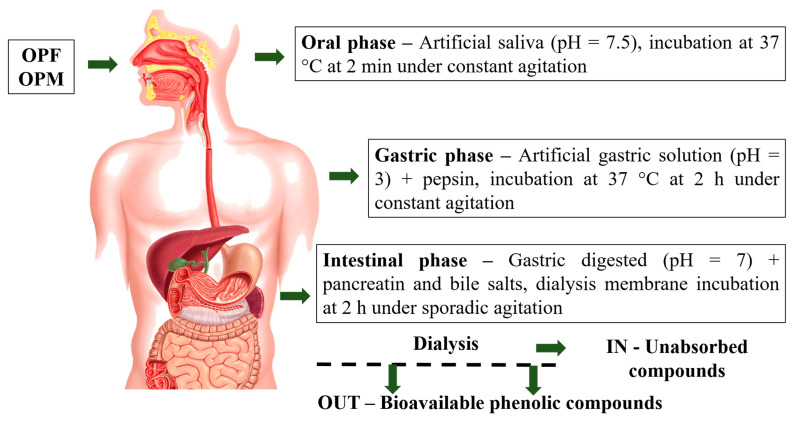
Olive pomace gastrointestinal digestion process steps. OPF—fractionated olive pomace (<2 mm) and OPM—fractionated and micronized olive pomace (<20 µm).

OPF and OPM olive pomace samples were submitted to the gastrointestinal digestion process following three sequential steps (Figure 1): (1) mouth digestion, (2) gastric digestion (stomach), and (3) duodenal digestion (small intestine), according to the in vitro international digestion protocol [17]. The mouth digestion step was simulated using artificial saliva (pH = 7.5) incubated at 37 °C for 2 min under constant agitation. At the end of the mouth digestion step, the pH of the digested sample was adjusted to pH 3.0 by the addition of an artificial gastric solution containing electrolytes (K^+^, Na^+^, Cl^−^, H_2_PO_4_^−^, HCO_3_^−^, CO_3_^2−^, Mg_4_^+^, NH_4_^+^, and Ca_2_^+^). Then, the digested sample was submitted to gastric digestion via incubation (37 °C for 2 h under agitation) and the addition of pepsin (2000 U mL^−1^). Subsequently, the pH of the gastric digested sample was adjusted to 7.0 using 1 M NaOH and submitted to intestinal digestion through the addition of pancreatin (100 U mL^−1^) and bile salts (10 mM). The final digested sample was placed into dialysis membranes (12000 Da, Sigma Aldrich, São Paulo, Brazil) immersed in 200 mL of phosphate buffer (24.96 mM, pH 7.2) and incubated at 37 °C for 2 h under sporadic agitation. At the end of intestinal digestion, two fractions, IN and OUT, were collected. The IN fraction was the one retained inside the dialysis membrane (corresponding to the digested fraction that remains in the small and large intestines), whereas the OUT fraction was the fraction able to be dialyzed, representing the bioavailable PCs from the OPF and OPM olive pomace samples. The bioavailable PCs from olive pomace were subjected to the evaluation of antioxidant and anti-neuroinflammatory ability in a cell-free system and microglial cells, respectively.

### 2.3. The Profile of Bioavailable PCs from Olive Pomace

According to [16], the bioavailable PCs in the OPF and OPM samples were extracted using an acidified acetone solution (0.35% formic acid, *v*/*v*; 7 mL). The phenolic extracts obtained were identified and quantified using an ultra-high-performance liquid chromatograph (Nexera XR, Shimadzu, Kioto, Japan) coupled to a triple-quadrupole mass spectrometer (UHPLC-MS/MS), according to [16]. Samples were injected (10 μL) in a C18 analytical column (4.6 mm × 150 mm, 1.8 μm particle size; Agilent Technologies, Santa Clara, CA, USA) at 35 °C. The mobile phase was HPLC-grade water with 0.5% acetic acid (eluent A) and acetonitrile (eluent B) at 0.2 mL min^−1^, and the chromatographic separation was performed in reverse-phase mode. Analytical curves were constructed using commercial standards of verbascoside (Chromadex, Los Angeles, C18, CO, USA), protocatechuic acid, 3-hydroxytyrosol, 4-hydroxybenzoic acid, tyrosol, caffeic acid, vanillic acid, homovanillic acid, *p*-coumaric acid, ferulic acid, oleuropein, luteolin, and apigenin (Sigma-Aldrich, St. Louis, MO, USA). PCs were quantified using authentic reference standards except for hydroxytyrosol-glycoside, which was quantified as an equivalent of hydroxytyrosol, and oleuropein aglycone, which was quantified as an equivalent of oleuropein [16]. The experiments were performed by setting the concentration of bioavailable PCs in OPF and OPM samples at 0.03, 0.15, 0.30, 1.5, and 3 mg L^−1^.

The validation of the methodology, as well as the standard curves and validation for the quantification of bioavailable PCs from olive pomace, have already been determined by our research group. This information is available and described in [16], and the limits of detection (LOD) and quantification (LOQ) for phenolic acids ranged from 0.0001 to 0.0012 mg L^−1^ and 0.0004 to 0.0035 mg L^−1^; for phenolic alcohols, they ranged from 0.0006 mg L^−1^ to 1.1607 mg L^−1^ and 0.0019 to 3.5172 mg L^−1^; and for secoiridoid compounds, they were 0.0004 mg L^−1^ and 0.0013 mg L^−1^, for LOD and LOQ, respectively.

### 2.4. In Vitro Antioxidant Capacity of Bioavailable PCs from Olive Pomace

The bioavailable PCs from OPF and OPM samples were analyzed for their antioxidant potential against hydroxyl radical (^●^OH) generation, ability to protect against glutathione (GSH) oxidation, and ability to protect against the peroxyl (ROO^●^) radical using the methods described below [18,19,20].

#### 2.4.1. Hydroxyl Radical Generation

The ability of a sample to remove the ^●^OH radical generated via the Fenton reaction is evaluated in the deoxyribose assay [19]. The bioavailable PCs from olive pomace (0.03, 0.15, 0.30, 1.50, and 3 mg L^−1^) were incubated for 1 h at 37 °C in test tubes containing potassium phosphate buffer (TFK, 50 mM), ethylendiaminetetraacetic acid (EDTA, 1 mM), iron chloride (FeCl_3_, 1 mM), water, ascorbic acid (2 mM), deoxyribose (60 mM), and hydrogen peroxide (H_2_O_2_, 10 mM). To make pink or orange compounds, we added 1 mL of thiobarbituric acid (TBA) and 1 mL of hydrochloric acid (HCl, 25%) to each tube. Then, we put the tubes in a water bath at 100 °C for 15 min. The reduction in the intensity of the pink or orange color caused by the samples was measured using a spectrophotometer at 532 nm, and the results were expressed as a percentage of ^●^OH radical generation [19].

#### 2.4.2. GSH Protection Capacity

The GSH assay was used to see if bioavailable PCs from olive pomace could prevent GSH oxidation and the reduction in sulfhydryl groups caused by H_2_O_2_ [18]. The samples were incubated (0.03, 0.15, 0.30, 1.50, and 3 mg L^−1^) for 1 h in test tubes containing TFK (1 mM), water, H_2_O_2_ (5 mM), and GSH (6 mM). At the end of the incubation, the measurement of the reduction in the sulfhydryl groups from GSH was carried out using 5,5 dithio bis-(2-nitrobenzoic acid) (DTNB). The results were expressed in GSH content (nmol GSH mL^−1^ of DTNB) [18].

#### 2.4.3. Protective Capacity against the Radical ROO^●^

The Oxygen Radical Absorbance Capacity (ORAC) method measures the scavenging capacity of a compound against the peroxyl radical, generated through 2,2-azobis-2-methylpropanoamidine (AAPH) at 37 °C. In 96-well plates, 25 µL of bioavailable PCs from olive pomace, previously diluted (100 times) in potassium phosphate buffer (75 mM) and 150 µL of fluorescein working solution (81 nM), was added. The plate was incubated for 10 min at 37 °C, for the last 3 min under constant agitation. After the incubation period, 25 µL of the AAPH solution was added (152 mM). Monitoring of fluorescence decay was followed in a fluorescence reader (Enspire 2300, Multimode Plate Reader, Perkin Elmer, Waltham, MA, USA) at 37 °C for 90 min. Excitation and emission wavelengths of 485 nm and 528 nm, respectively, were used. Results were expressed as µmol of Trolox equivalents per mL of sample [20].

### 2.5. Evaluation of the Anti-Neuroinflammatory Capacity of Bioavailable PCs from Olive Pomace in Microglial Cells

#### 2.5.1. Cell Culture and Treatments

The microglial cell line BV-2 (ATCC^®^CRL-2467TM) was purchased from the Cell Bank of Rio de Janeiro (the “Banco de Células do Rio de Janeiro”, BCRJ, Rio de Janeiro, RJ, Brazil). The cells were cultured in RPMI 1640 cell culture medium (Sigma-Aldrich, #R8758, São Paulo, SP, Brazil). The cells were kept in a CO_2_ incubator under ideal conditions for cell culture (5% CO_2_ at 37 °C). The culture medium was supplemented with 10 mM of HEPES, 10% of fetal bovine serum (FBS) (Sigma-Aldrich, #F2442, São Paulo, SP, Brazil), and 1% penicillin (100 U mL^−1^)/streptomycin (100 mg mL^−1^) (Sigma-Aldrich, #P4333, São Paulo, SP, Brazil). Treatments were conducted on BV-2 cells after 6–8 passages [6].

BV-2 cells were seeded in 96-well plates (density of 2.5 × 105 cells mL^−1^ per well) and treated with a concentration-response curve of bioavailable PCs from OPF and OPM samples (0.03, 0.15, 0.30, 1.50, and 3 mg L^−1^) for 24 h to evaluate the effect of the treatments through cell proliferation and analysis of NO production. Hydrogen peroxide (H_2_O_2_) (100 μM) was used as a positive control for cell death induction [21].

In a second moment, BV-2 cells were treated with 1 μg mL^−1^ after exposure to LPS, and activated cells were treated with the bioavailable PCs from OPF and OPM samples (0.03, 0.15, 0.30, and 1.50 mg L^−1^) for an additional 24 h to verify the ability of the samples to reduce the inflammatory response. The anti-neuroinflammatory capacity was evaluated through the determination of cell viability and the measurement of levels of ROS and RNS [1].

#### 2.5.2. Cellular Viability Determination

The viability of BV-2 microglial cells inactivated or activated with LPS and exposed to the samples of this study was determined using MTT bromide (3-(4,5-Dimethyl-2-thiazolyl-2,5-diphenyl-2H-tet-razonium) reagent (Sigma-Aldrich-M2128; St. Louis, MO, USA). After the incubation period with the bioavailable PCs from the OPF and OPM samples, the treatments were removed, and the cells were resuspended in phosphate buffer (PBS) 1×, adding 20 μL of 5 mg mL^−1^ MTT. After incubation at 37 °C for 2 h, the intracellular formazan crystals were solubilized using demethylsulfoxide (DMSO). Absorbance was determined at 570 nm using a plate reader (Biochrom^®^ Anthos 2010, London, UK) [22].

#### 2.5.3. Measurement of NO Production

NO levels were determined through an indirect method based on the use of the Greiss reagent (1% sulfanilamide + 0.1% N-(1-naphthyl)ethylenediamine dihydrochloride) [23]. After activation by LPS and treatment with the bioavailable PCs from OPF and OPM samples, the cell supernatant was added to the Greiss reagent and analyzed. The Greiss reagent can detect organic nitrite (NO metabolite). The sulfanilamide in this reagent interacts with the nitrite in the sample, forming diazonium salts that turn pink or purple when in contact with N-(naphthyl 1-dihydrochloride) ethylenediamine. The staining intensity is directly proportional to the NO production. Absorbance was measured at 540 nm using a plate reader (Biochrom^®^Anthos 2010, London, UK) [23].

#### 2.5.4. Measurement of ROS Levels

Total ROS levels in LPS-inactivated and -activated cells after treatment with the bioavailable PCs from OPF and OPM samples were determined using DCFH-DA (2′ Dichlorodihydrofluorescein 2′ diacetate) (Sigma Aldrich-D6883; Sao Paulo, SP, Brazil) [24]. DCFH-DA is metabolized by intracellular enzymes, forming dichlorodihydrofluorescein (DCFH). ROS can reduce DCFH into dichlorofluorescein (DCF). DCF emits fluorescence that can be measured at 525 nm excitation and 488 nm emission. The fluorescence intensity was measured using a plate reader (Spectra Max i3, Molecular Devices, San Jose, CA, USA) [24].

### 2.6. Statistical Analyses

All experiments were repeated at least three times. The results were expressed as the mean ± standard error of the mean (SEM). All data analyses were performed using Graph Pad Prism Software version 5.0 (La Jolla, CA, USA), and differences were considered significant when *p* < 0.05, evaluated by one-way analysis of variance (ANOVA) followed by the Tukey test.

To investigate the association between HPLC-MS assessment of PCs and the anti-neuroinflamatory effect, chemometric analyses such as principal component analysis (PCA) and cluster analysis (CA) were carried out. The CA was performed for treatments using the Distance, Cluster, and Trace procedures, using the average Euclidean distance as a dissimilarity measure and Ward as a clustering method. PCA was performed using the PRINQUAL, PRINCOMP, and FACTOR procedures (Khattree and Naik 2000). MANOVA was performed on the SAS^®^ System for Windows™ version 9.4 (SAS Institute Inc., Cary, NC, USA) at a 5% significance level.

## 3. Results

### 3.1. The Profile of Bioavailable PC in OPF and OPM Samples

The total concentration of the bioavailable PCs in the OPM sample was higher than that observed in the OPF sample (Table 1, *p* < 0.05). The main groups of PCs present in olive pomace are flavonols, secoiridoids, phenolic acids, phenolic alcohols, and lignins. The phenolic alcohols found in the bioavailable fractions were hydroxytyrosol-glucoside, hydroxytyrosol, and tyrosol, with hydroxytyrosol-glucoside being the most representative in both samples. OPM showed higher concentrations of hydroxytyrosol-glucoside when compared with OPF (Table 1, *p* < 0.05). This same profile was observed for secoiridoid compounds, represented by oleuropein aglycone, 4-hydroxybenzoic acid, and chlorogenic acid, where OPM presented a higher content than OPF (Table 1, *p* < 0.05).

### 3.2. In Vitro Cell-Free Antioxidant Capacity of the Bioavailable PCs from OPF and OPM Samples

The bioavailable PCs from olive pomace did not show antioxidant potential (*p* ≥ 0.05) against the ^●^OH radical and H_2_O_2_ regardless of the concentration evaluated and the particle size of the olive pomace. However, the bioavailable PCs from olive pomace showed antioxidant capacity against the ROO^●^ radical, and this effect was higher for the OPM sample when compared with OPF (4.30 µM Trolox mL^−1^ vs. 3.31 µM Trolox mL^−1^, *p* < 0.05).

### 3.3. Anti-Neuroinflammatory Capacity of the Bioavailable PCs from Olive Pomace in Microglial Cells

**Figure 2:**
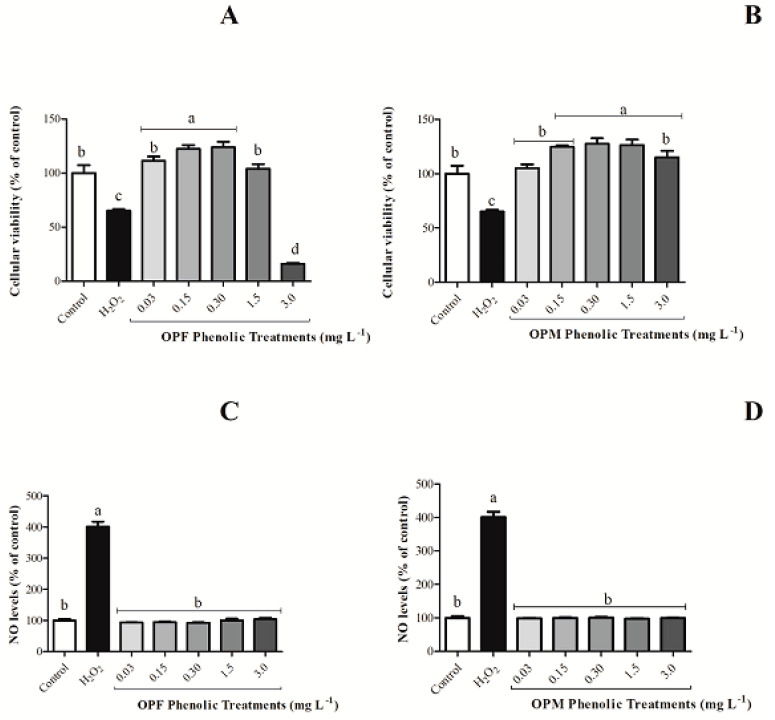
Per se effect of bioavailable PCs from OPF and OPM samples (0.03, 0.15, 0.30, 1.5, and 3 mg L^−1^) on cell viability (**A**,**B**) and on NO levels (**C**,**D**) in BV-2 microglial cells after 24 h of treatment. Results are shown in percentages and presented as mean ± standard error of mean (SEM), and different letters indicate significant differences between groups. Statistical analysis was carried out via one-way ANOVA followed by Tukey post hoc. Results with *p <* 0.05 were considered significant.

As expected, H_2_O_2_ decreased the cellular viability and increased NO production when compared with the negative control. The OPF sample, at the highest evaluated concentration (3 mg L^−1^), reduced per se the cell viability of BV-2 cells by 83.82% (Figure 2A, *p* < 0.05). This behavior was not observed for the OPM sample (Figure 2B, *p* > 0.05). Additionally, all the OPM evaluated concentrations were shown to be safe, since none of the concentrations caused cellular mortality (Figure 2B). Also, none of the olive pomace samples induced increased NO levels compared with the negative control by itself (Figure 2C,D). Thus, the concentration of 3 mg L^−1^ was not evaluated for the anti-neuroinflammatory capacity of olive pomace samples in BV-2 cells activated by LPS.

**Figure 3:**
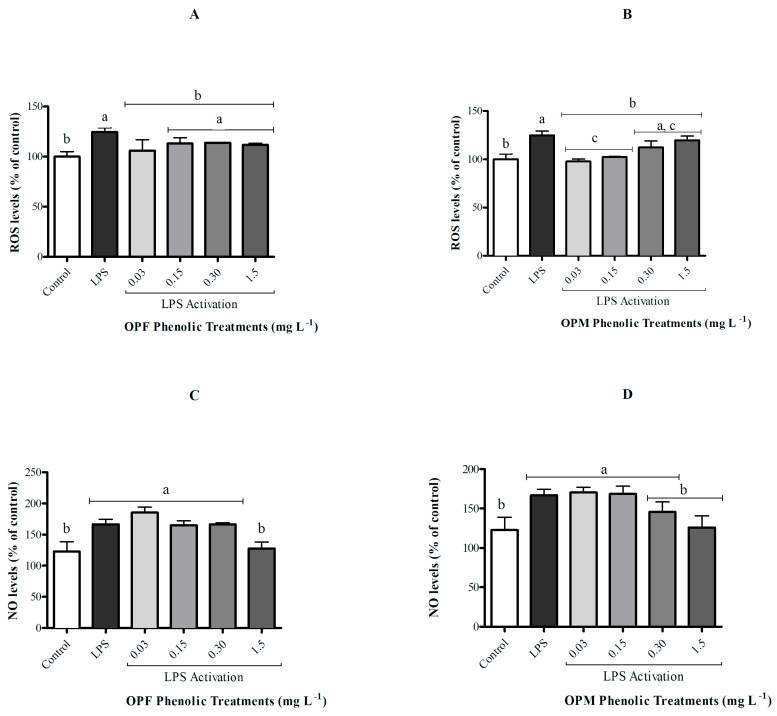
Effect of bioavailable PCs from the OPF and OPM samples (0.03, 0.15, 0.30, and 1.5 mg L^−1^) on the ROS (**A**,**B**) and NO levels (**C**,**D**) on LPS-activated (1 µg mL^−1^) microglial cells after 24h of treatment. Results are shown in percentages and presented as mean ± standard error of mean (SEM), and different letters indicate significant differences between groups. Statistical analysis was carried out via one-way ANOVA followed by Tukey post hoc. Results with *p* < 0.05 were considered significant.

The activation of microglial cells by LPS did not affect cell viability although it resulted in an increase in ROS and NO levels when compared with the control group (Figure 3, *p* < 0.05). The OPF sample was able to significantly attenuate ROS generation in activated microglial cells only at its lowest concentration (0.03 mg L^−1^), while the OPM sample presented a protective effect of reducing ROS levels at both 0.03 and 0.15 mg L^−1^ (Figure 3A,B *p* < 0.05). In contrast, only the highest concentration of PCs (1.5 mg L^−1^) from both OPF and OPM samples was able to significantly attenuate (approximately 25%) the increase in NO levels induced by LPS in BV-2 cells (*p* < 0.05, Figure 3C,D).

### 3.4. Chemometric Analyses to Identify the Bioactive PCs of Olive Pomace

The relationship between PCs from olive pomace and the anti-neuroinflammatory effect was investigated using MANOVA. MANOVA using the Wilks, Pillai, Hotelling–Lawley, and Roy tests revealed that when all variables were analyzed together, there was a significant interaction effect (types of processing × inclusion levels).

**Figure 4:**
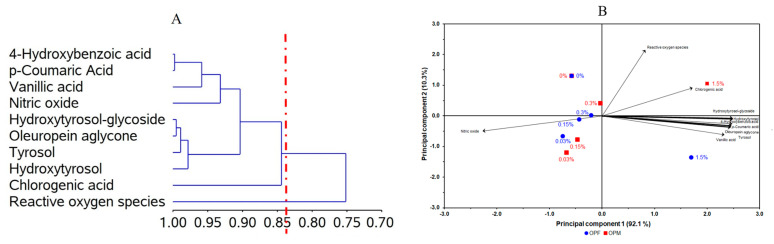
Dendrogram of polyphenols, anti-inflammatory, and antioxidant activities (ordinate axis) in relation to the coefficient of determination (r^2^; abcissa axis) using the correlation matrix as similarity measure and principal component as clustering method (**A**). Bidimensional biplot from olive pomace (OP) under different processing types (OPF = fractioned, OPM = micronized) and inclusion levels (0.03, 0.15, 0.30, and 1.50 mg L^−1^) versus polyphenols, anti-inflammatory, and antioxidant activities (loadings) in relation to the principal components (**B**). (**A**) = 84.4% and (**B**) = 92.1%.

The CA of bioavailable PCs revealed the formation of two distinct groups, which were able to explain 84.4% of the total data variation (Figure 4A). One of the groups includes chlorogenic acid, hydroxytyrosol, tyrosol, oleuropein aglycone, hydroxytyrosol-glucoside, nitric oxide, vanillic acid, *p*-coumaric acid, and 4-hydroxybenzoic acid. This grouping suggests that the NO levels were first influenced by vanillic, *p*-coumaric, and 4-hydroxybenzoic acids.

The PCA was used as an exploratory analysis to verify whether the distinct types of olive pomace processing and their different concentrations affected the anti-neuroinflammatory capacity of bioavailable PCs from olive pomace (Figure 4B). A two-dimensional biplot of different processing types and different concentrations vs. PC, NO, and ROS levels presented a proportion of explained variance of 92.1% of the data. According to the PCA (Figure 4B), we can see that, for both samples, lower concentrations (0.03, 0.15, and 0.30 mg L^−1^) were associated with a high antioxidant potential (reduced ROS levels) and a low anti-neuroinflammatory effect (higher NO levels). On the other hand, at higher concentrations (1.5 mg L^−1^) of bioavailable PCs from olive pomace, lower NO levels were observed, suggesting an anti-neuroinflammatory effect, while less potential for reducing ROS levels was observed.

### 3.5. Distribution of Bioavailable PC Classes from OPF and OPM

**Figure 5:**
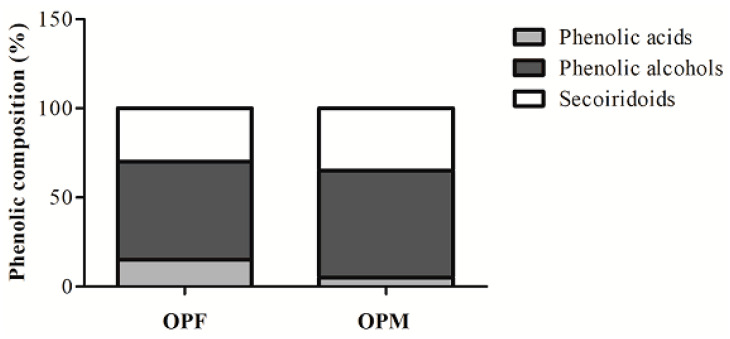
Changes in the distribution of bioavailable phenolic compound classes from fractionated (OPF) and micronized (OPM) olive pomace.

The ability of OPF and OPM to reduce NO and ROS levels may likely be associated with the PCs presented in the samples. The distribution of different classes of bioavailable PCs in OPF and OPM olive pomace is reported in Figure 5. The OPF presents in its composition 15% phenolic acids (4-hydroxybenzoic, vanillic, and *p*-coumaric acids), 55% phenolic alcohols (hydroxytyrosol, hydroxytyrosol–glycoside, and tyrosol), and 30% secoiridoid compounds (oleuropein aglycone). On the other hand, OPM presented a different distribution, where only 5% of the bioavailable PCs were represented by phenolic acids (4-hydroxybenzoic, vanillic, *p*-coumaric, and chlorogenic acids). Phenolic alcohols and secoiridoid compounds were in greater proportions in the OPM sample, at 60% and 35%, respectively.

## 4. Discussion

Most of the PCs present in olive pomace are rapidly metabolized and absorbed in the gastrointestinal tract [14,25]. The in vitro gastrointestinal digestion process of the OPF and OPM samples resulted in two fractions, the IN and OUT fractions. The IN fraction is retained on the dialysis membrane and corresponds to the digested fraction that remains in the colon for subsequent colonic fermentation. The OUT fraction is the one that permeates the dialysis membrane and corresponds to the bioavailable fraction of the digested sample [26]. In this study, our interest was focused on the OUT fractions of the OPF and OPM samples, which correspond to the bioavailable PCs that perform different protective functions in the human body [27].

The amount of the bioavailable PCs in the OPM sample was higher than that observed in the OPF sample, and this result may be associated with the particle size of the samples, where the smallest particle size (OPM) results in a greater contact surface with the enzymes involved in the gastrointestinal digestion process, resulting in a greater extraction of PCs [16]. These results corroborate those presented by other researchers, where hydroxytyrosol, hydroxytyrosol glycoside, tyrosol, apigenin, and *p*-coumaric acid were identified in the bioavailable fraction of olive pomace after the in vitro digestion process [16,26,28]. Oleuropein is the most representative secoiridoid compound in olive pomace, followed by phenolic alcohols, tyrosol, and hydroxytyrosol. A large variety of phenolic acids are present in olive pomace, especially vanillic, caffeic, *p*-coumaric, ferulic, and gallic acids. Pinoresinol and its derivatives are the most common lignins in olive pomace, while rutin, apigenin, and luteolin are the most representative flavonol compounds [14,29]. In agreement with the results above, the bioavailable PCs from OPM had a higher antioxidant capacity against peroxyl radical than the ones from OPF. This characteristic of the bioavailable PCs from olive pomace can be helpful in the mediation of oxidative and neuroinflammatory processes [6,14,26].

In the present study, the bioavailable PCs of olive pomace acted as neuroprotectors by attenuating neuroinflammatory processes. The digestive process is important in defining PC biological properties, as it causes significant changes in the PC profile in foods [14]. Throughout the digestion stages, PCs may undergo changes in their structures, due to changes in pH. These alterations result in an increased reactivity and, consequently, a possible increase in the bioactivity of the PCs present in the food matrix [28,30]. In fact, of the total ingested PCs, it is estimated that only 10%, at the end of the digestive process, remain bioavailable to play their protective role in the body [14].

The solubility characteristic of a PC is what determines its permeability across the blood–brain barrier (BBB), where less polar compounds (i.e., O-methylated derivatives) are more permeable when compared with more highly polar PCs (i.e., sulfated and glucuronidated derivatives) [31]. This peculiarity of the PCs in olive oil and olive pomace makes them compounds with potential for the treatment of neuroinflammation [32]. The ability to cross the BBB has already been described for PCs from several sources [33,34,35]. Moreover, the bioavailability of PCs as epigallocatechin gallate, epicatechin, and anthocyanins after oral administration in the brain is low (less than 1 nmol g^−1^). However, even at low concentrations, they play an important role in neuroprotection through the regulation of pro-inflammatory genes [31].

In fact, the OPM sample presented a higher amount of bioavailable PCs and antioxidant in vitro activity than the OPF. However, all evaluated concentrations were correct based on their phenolic composition, so both OPF and OPM contain the same amount of PCs. Nonetheless, OPM exhibits a distinct distribution of PC subclasses in comparison with OPF. Thus, the present study shows that the different proportions of bioavailable PCs in the OPF and OPM samples may directly affect their potential to reduce ROS and NO levels, since PCs may act synergistically [14]. That could explain the lack of differences among OPF and OPM on ROS and NO levels in cells, as revealed via the PCA. Moreover, the PCA also revealed that higher concentrations of bioavailable PCs from olive pomace are needed for the anti-neuroinflammatory potential when compared with those required for the antioxidant effects in activated microglia cells, independently of the particle size evaluated. We suggest that this fact may be associated with oxidative stress induced by neuroinflammation, which, as it is an event that involves different factors, results in the need for higher concentrations of PCs. However, regarding the antioxidant effect, we highlight that lower concentrations of PCs can be beneficial, since we demonstrated for the first time in our study that higher concentrations of PCs from olive pomace can induce cytotoxicity, which may be the result of a possible pro-oxidant effect at higher concentrations.

The reactivity of PCs to ROS depends on their structures, which may imply different complementary mechanisms where PCs act simultaneously [36]. Thus, we cannot rule out that the observed effects may be a result of the proportion and distribution of PCs in each analyzed sample rather than the amount of a specific compound. Accordingly, the combination of PCs in olive oil (equimolar proportion of oleuropein, tyrosol, and *p*-coumaric acid) was more efficient in delaying neuronal death and combating oxidative stress and neuroinflammation associated with neurodegenerative diseases [36].

The CA suggests that NO levels were first influenced by vanillic, *p*-coumaric, and 4-hydroxybenzoic acids. In fact, the OPF sample had a higher phenolic acids content than the OPM. Accordingly, *p*-coumaric acid (80 mg kg^−1^ bw) is able to inhibit the generation of pro-inflammatory mediators induced by LPS, especially through its antioxidant action and ability to inhibit cytokine production [37]. This phenolic acid (75 mg kg^−1^ bw) also reduced the expression of pro-inflammatory cytokines (IL-1β and TNFα), protecting against depression and memory loss in a mouse model of depression induced by costicosterone [38]. Similarly, vanillic acid (80 mg kg^−1^ bw) can reduce oxidative stress induced by β-amyloid peptide (Aβ_1-42_), which demonstrates its neuroprotective role [39]. Furthermore, chlorogenic acid (4 mg kg^−1^ bw) was able to inhibit the pro-inflammatory pathway in BV-2 cells of male C57BL/6 mice activated by LPS and promote their polarization to the anti-neuroinflammatory pathway [40].

On the other hand, OPM presented a higher proportion of phenolic alcohols and secoiridoid compounds than OPF. Oleuropein and its main metabolite, hydroxytyrosol, are the main PCs in olive oil and pomace and have been widely studied, especially because of their easy administration through food [32]. Isolated hydroxytyrosol and oleuropeín presented antioxidant and anti-inflammatory effects in microglial cells activated by LPS [12,32] or α-synuclein [11]. One of the main PCs from olive pomace, hydroxytyrosol (50–200 mg kg^−1^ bw), alleviates oxidative stress and neuroinflammation, as well as enhances hippocampal neurotrophic signaling in a depression model in mice [36].

PCs from oil and olive pomace are recognized for their antioxidant and neuroprotective properties but, in most cases, they are studied individually. One study has shown that whole extra-virgin olive oil, which is rich in oleuropein, tyrosol, and hydroxytyrosol, was able to reduce the production of inflammatory mediators and suppress the secretion of pro-inflammatory cytokines in BV-2 microglial cells activated by LPS [34]. Furthermore, the Mediterranean diet, characterized by a high consumption of olive oil, highlights the importance of olive PCs and the different health benefits that are linked to the consumption of these compounds [1]. Although studies on the beneficial potential of PCs from olive pomace are still scarce, our study is a pioneer in describing some of the benefits of this by-product. Thus, we highlight the importance of further studies aimed at guaranteeing the biological properties of the PCs present and the food safety of olive pomace as a diet enricher, in addition to studies on possible food applications.

Currently, olive pomace is considered a problem for the olive oil industry, as there is no specific purpose for this by-product. Although olive pomace can be used in animal feed, soil fertilization, and energy generation, these uses still do not meet the demands of industries [12]. Furthermore, the use of olive pomace can increase the sustainability of the olive oil industry and, in turn, lead to relevant economic benefits [12]. In this study, we showed the promising role of PCs from olive pomace in the prevention and mediation of oxidative and neuroinflammatory processes in microglia cells. Thus, this bioactivity may improve the range of olive pomace applications.

Moreover, we reported for the first time that the OPF sample at the highest evaluated concentration (3 mg L^−1^) reduced the cell viability of BV-2 cells per se. These results indicate that high concentrations of OPF may be toxic to microglial cells. We can propose that this toxic effect of the higher concentration of OPF was possibly due to the lower concentrations of the compounds hydroxytyrosol-glycoside, 4-hydroxybenzoic acid, and oleuropein aglycone, which act synergistically with the others. This same toxic potential was not observed for OPM, where the concentrations of hydroxytyrosol-glycoside, 4-hydroxybenzoic acid, and oleuropein aglycone were higher and may have acted synergistically with the other compounds, not resulting in a toxic potential for microglial cells. Therefore, we highlight that it is always important to consider the distribution of PCs in different concentrations in a complex matrix, such as food.

## 5. Conclusions

This study shows, for the first time, that bioavailable PCs from olive pomace present antioxidant potential in cell-free systems and anti-neuroinflammatory potential in microglia cells. The antioxidant and the anti-neuroinflammatory effects are most likely associated with the distribution of bioavailable PCs in olive pomace samples and their synergistic action. We demonstrated that bioavailable PCs from olive pomace are a promising option for combating diseases associated with neuroinflammation, regardless of particle size. However, studies on its possible toxic effects and safe concentrations are essential for its nutritional application. We highlight the latter as one of the main gaps to be filled to understand the actual health benefits of the PCs present in olive pomace. Olive pomace is considered a problem for the olive oil production industry, as there is no specific purpose for this by-product. In this study, we showed the promising role of PCs from olive pomace in the prevention and mediation of oxidative and neuroinflammatory processes, highlighting their possible food application. Furthermore, the use of olive pomace can increase the sustainability of the olive oil industry and, in turn, lead to relevant economic benefits.

## Figures and Tables

**Table 1:** Bioavailable PCs (mg L^−1^) of fractionated (OPF) and micronized (OPM) olive pomace.

Phenolic Compounds	OPF	OPM
Hydroxytyrosol	0.10 ± 0.02 ^a^	0.18 ± 0.04 ^a^
Hydroxytyrosol–glycoside	4.53 ± 0.08 ^b^	8.07 ± 0.60 ^a^
4-Hydroxybenzoic Acid	0.008 ± 0.002 ^b^	0.015 ± 0.00 ^a^
Tyrosol	1.15 ± 0.07 ^a^	1.56 ± 0.31 ^a^
Chlorogenic Acid	<LOQ	0.01 ± 0.00 ^a^
Vanillic Acid	0.13 ± 0.04 ^a^	0.12 ± 0.05 ^a^
Oleuropein aglycone	4.65 ± 0.07 ^b^	5.95 ± 0.12 ^a^
*p*-Coumaric Acid	0.063 ± 0.01 ^a^	0.093 ± 0.01 ^a^
Oleuropein	<LOQ	<LOQ
Total PC content	10.65 ± 0.04 ^b^	16.02 ± 0.35 ^a^

Results are presented as mean ± standard error of mean (SEM), and different letters indicate significant differences (*p* < 0.05) via one-way ANOVA and Tukey test. LOQ: limit of quantification.

## Data Availability

The data presented in this study are available on request from the corresponding author.
